# Small-angle neutron scattering reveals the assembly mode and oligomeric architecture of TET, a large, dodecameric aminopeptidase

**DOI:** 10.1107/S1399004714018446

**Published:** 2014-10-23

**Authors:** Alexandre Appolaire, Eric Girard, Matteo Colombo, M. Asunción Durá, Martine Moulin, Michael Härtlein, Bruno Franzetti, Frank Gabel

**Affiliations:** a Université Grenoble Alpes, IBS, 38044 Grenoble, France; b CNRS, IBS, 38044 Grenoble, France; c CEA, IBS, 38044 Grenoble, France; d Life Sciences Group, Institut Laue–Langevin, 38042 Grenoble CEDEX 9, France; e Large Scale Structures Group, Institut Laue–Langevin, 38042 Grenoble CEDEX 9, France

**Keywords:** TET, small-angle neutron scattering

## Abstract

The specific self-association of proteins into oligomeric complexes is a common phenomenon in biological systems to optimize and regulate their function. However, *de novo* structure determination of these important complexes is often very challenging for atomic-resolution techniques. Furthermore, in the case of homo-oligomeric complexes, or complexes with very similar building blocks, the respective positions of subunits and their assembly pathways are difficult to determine using many structural biology techniques. Here, an elegant and powerful approach based on small-angle neutron scattering is applied, in combination with deuterium labelling and contrast variation, to elucidate the oligomeric organization of the quaternary structure and the assembly pathways of 468 kDa, hetero-oligomeric and symmetric *Pyrococcus horikoshii* TET2–TET3 aminopeptidase complexes. The results reveal that the topology of the *Ph*TET2 and *Ph*TET3 dimeric building blocks within the complexes is not casual but rather suggests that their quaternary arrangement optimizes the catalytic efficiency towards peptide substrates. This approach bears important potential for the determination of quaternary structures and assembly pathways of large oligomeric and symmetric complexes in biological systems.

## Abstract 2

The present work illustrates that small-angle neutron scattering, deuteration and contrast variation, combined with *in vitro* particle reconstruction, constitutes a very efficient approach to determine subunit architectures in large, symmetric protein complexes. In the case of the 468 kDa heterododecameric TET peptidase machine, it was demonstrated that the assembly of the 12 subunits is a highly controlled process and represents a way to optimize the catalytic efficiency of the enzyme.

## 1. Introduction   

The specific self-association of proteins to form oligomeric machines is a common phenomenon in biological systems. Homo-oligomerization in a closed symmetry is particularly prevalent in enzymes and high-order oligomers comprising more than six subunits and represents a significant part of the proteomes (Matthews & Sunde, 2012 ▶). There are numerous reasons why enzymes self-assemble into large edifices: while representing a way to increase the stability and solubility of the system, the main advantage afforded by complex quaternary structures is to improve enzymatic function. Subunit interactions often allow the formation of channels to increase the specific affinity towards the substrate(s). Oligomerization also induces cooperativity between monomers to build up active sites or to share them at interfaces (Marianayagam *et al.*, 2004 ▶) and, in multi-subunit enzymatic complexes, the quaternary structure enhances functional cooperativity (Griffin & Gerrard, 2012 ▶). Monomers assembled into large, hollow edifices can create compartments for the confinement of biochemical activities. In the case of energy-dependent chaperonins or complexes of ATPases associated with various cellular activities (AAA-ATPases), this strategy is exploited to unfold, refold or disassemble macromolecular edifices in the interior of a nano-compartment, thus avoiding aggregation in the crowded cytosolic environment (Snider & Houry, 2008 ▶). In the case of large intracellular peptidases such as proteasomes, the self-compartmentalization allows the confinement of the active sites inside the final particle and, in this way, uncontrolled proteolytic activity is avoided in the cytosol (Sauer & Baker, 2011 ▶). Finally, another advantage of oligomerization is to generate docking platforms for regulatory proteins or complexes. For all of these reasons, large enzymatic machines must adopt a well defined quaternary structure to carry out their function, and one way to regulate their activity within the cell is by controlling their oligomerization state.

Surprisingly, only a few studies have addressed the fundamental questions of the assembly pathways of large homo-oligomers. Progress has been made in recent years, indicating that dimeric precursors are often involved (Marianayagam *et al.*, 2004 ▶). However, the structural details and control mechanisms of the assembly processes are still largely unknown: are they orchestrated, stepwise processes with well defined oligomeric intermediates? Does assembly follow a limited number of pre-defined pathways or is it of a random nature? How are the intermediate oligomers positioned in the final complex? Concomitantly, the study of the assembly processes of homomeric, symmetric complexes poses two practical challenges: (i) the isolation, stabilization and biochemical/biophysical characterization of the building blocks and intermediate states and (ii) the determination of their respective positions and arrangement within the final edifice. Here, we apply a powerful and elegant approach based on the combination of small-angle neutron scattering (SANS), deuterium labelling and contrast variation to elucidate the oligomeric organization of the quaternary structures and the assembly modes of symmetric, heterododecameric, 468 kDa TET aminopeptidase complexes.

TET complexes are bi-metallic aminopeptidases that act as peptide-destruction machines and are widespread in the three domains of life. They belong to the M42 and M18 families in clan MH according to the MEROPS classification system (Rawlings *et al.*, 2014 ▶). The typical TET tetrahedral quaternary structure was initially described in archaea (Franzetti *et al.*, 2002 ▶; Russo & Baumann, 2004 ▶; Borissenko & Groll, 2005 ▶), but has also been found in bacteria (Kim *et al.*, 2010 ▶) and in eukarya (Chen *et al.*, 2012 ▶; Chaikuad *et al.*, 2012 ▶). Unlike most homomers, which adopt cyclic or dihedral symmetries, TET peptidases display unusually sophisticated quaternary structures, with 12 monomers being arranged in a hollow, tetrahedral edifice (Franzetti *et al.*, 2002 ▶). The crystallographic structures and the enzymatic properties of three different complexes (*Ph*TET1, *Ph*TET2 and *Ph*TET3) from the hyperthermophilic archaeon *Pyrococcus horikoshii* have been determined (Durá *et al.*, 2005 ▶, 2009 ▶; Schoehn *et al.*, 2006 ▶). They form self-compartmentalized assemblies with a common internal organization made up of a network of four access channels extended by four vast catalytic chambers, each containing three active sites close to the tetrahedron apices (Durá *et al.*, 2005 ▶). Each TET subunit is formed by a proteolytic and a PDZ-like domain that mediate both the dimeric interface and the dimer–dimer interface that build the whole tetrahedral particle. We recently demonstrated that the activity of TET aminopeptidase towards long polypeptides is coupled with its assembly process (Appolaire *et al.*, 2013 ▶; Rosenbaum *et al.*, 2011 ▶): the co-occurrence *in vivo* of stable TET dimeric precursors and assembled dodecamers suggests that, at least in archaea, the TET oligopeptidase activity is regulated by control of its oligomerization state. *Ph*TET homododecamers are very stable, even at high temperatures (90°C), a fact attributed to the properties of the dimer interfaces (Appolaire *et al.*, 2013 ▶). *In vivo* studies, combined with small-angle X-ray scattering (SAXS), showed that dimers are the elementary building blocks and, based on electron microscopy (EM) and analytical ultracentrifugation (AUC) data, a working model with hexameric intermediates assembling into the final dodecameric edifices has been suggested (Appolaire *et al.*, 2013 ▶).

The three M42 protein homomers comprised in the genome of *P. horikoshii* display complementary substrate specificities: *Ph*TET1 is a glutamyl-aminopeptidase that is active towards acidic residues, *Ph*TET2 is a leucyl-aminopeptidase that is active towards neutral residues, and *Ph*TET3 is a lysyl-aminopeptidase that is mainly active towards basic residues. The three forms therefore represent an integrated cellular peptide-destruction system (Durá *et al.*, 2009 ▶). *Ph*TET2 and *Ph*TET3 display a high sequence identity, are robust thermozymes and their quaternary structures resist harsh physicochemical treatments (Rosenbaum *et al.*, 2012 ▶). Their assembly process is metal-dependent and, *in vitro*, addition of a metal chelator agent combined with high-pH conditions is necessary to disassemble the complex into its stable dimeric precursors. These can spontaneously reform the tetrahedral edifice upon dialysis against a physiological assembly buffer containing cobalt (Rosenbaum *et al.*, 2011 ▶).

Here, we show that when mixed together in the assembly buffer *Ph*TET2 and *Ph*TET3 dimers can self-organize into 468 kDa heterododecameric complexes that possess the same quaternary structure as homododecameric TETs. Using a combination of SANS and contrast variation (variable H_2_O/D_2_O ratio in the solvent; Jacrot, 1976 ▶) on heterododecameric complexes of deuterated *Ph*TET2 (‘d*Ph*TET2’) and hydrogenated *Ph*TET3 (‘h*Ph*TET3’) allowed us to elucidate their respective quaternary architectures within the assembled hetero-complex. Our results demonstrate that TET complexes are built following a very limited number of specific and well defined pathways. Intriguingly, the resulting geometric arrangement of the two different building blocks within the heterododecameric complex is not casual but rather suggests a mechanism to optimize its catalytic properties towards peptide substrates. Our approach represents an elegant and attractive method to address structural questions in other challenging oligomeric systems composed of symmetric (or very similar) building blocks such as the proteasome. Finally, a better structural insight into the assembly mechanisms of large complexes is essential for preparing studies of the underlying regulatory mechanisms, which have not been intensively explored to date but might represent attractive targets for biomedical approaches and drug design.

## 2. Material and methods   

### 2.1. Expression and purification of hydrogenated *Ph*TET3 and deuterated *Ph*TET2   

Total protein extracts of *Escherichia coli* cells expressing the various recombinant hydrogenated *Ph*TET3 proteins (h*Ph*TET3) were purified as described by Durá *et al.* (2009 ▶). For SANS experiments, random-fractional deuteration of the wild type and a pentamutant (see below) *Ph*TET2 protein (d*Ph*TET2) was carried out in the ILL Deuteration Laboratory, Grenoble, France. Cells were grown at 30°C in minimal medium as described by Artero *et al.* (2005 ▶) using 85%(*v*/*v*) D_2_O and unlabelled glycerol as a carbon source. At an OD_600_ of about 10 for the wild type and of about 13 for the pentamutant construct, the high cell-density cultures were induced with 1 m*M* IPTG overnight. The final deuteration level was approximately 75% to yield a SANS contrast match point of 100% D_2_O. The resulting cell pellets were stored at −80°C until further use.

For purification, the pellets were thawed at room temperature and resuspended in 50 ml 50 m*M* Tris–HCl, 150 m*M* NaCl, 0.1% Triton X-100 pH 8.0. Next, 12.5 mg lysozyme (Euromedex), 2.5 mg DNase I grade II (Roche), 10 mg RNase (Roche), 50 mg Pefabloc SC (Roche) and 0.5 ml 2 *M* MgSO_4_ were added to the cell suspensions. Disruption of the cells was achieved by sonication in a Branson Sonifier 150 at 4°C. Five 30 s bursts at intensity 10 with intermediary pauses of 30 s were employed. The crude extract was then heated at 85°C for 15 min to eliminate most of the mesophilic proteins from the *E. coli* host and the lysates were clarified by centrifugation at 17 400*g* for 1 h. Supernatant concentrations were adjusted to 100 m*M* NaCl (d*Ph*TET2) or 250 m*M* NaCl (h*Ph*TET3), 20 m*M* Tris pH 7.5. The resulting extracts were loaded onto a 6 ml Resource Q column (GE Healthcare) previously equilibrated in 100 m*M* NaCl, 20 m*M* Tris–HCl pH 7.5. After washing with three column volumes (CV) of the equilibration buffer, bound proteins were eluted at 4 ml min^−1^ with a 20 CV linear salt gradient (0.1–0.35 *M* NaCl for d*Ph*TET2 or 0.25–0.45 *M* NaCl for h*Ph*TET3 in 20 m*M* Tris–HCl pH 7.5). For further purification, the proteins were loaded onto a Superose 6 size-exclusion column (GE Healthcare) equilibrated and run in 20 m*M* Tris–HCl, 150 m*M* NaCl pH 7.5.

### 2.2. De-oligomerization of native TET homododecamers and formation of heterododecamers   

**Figure 1:**
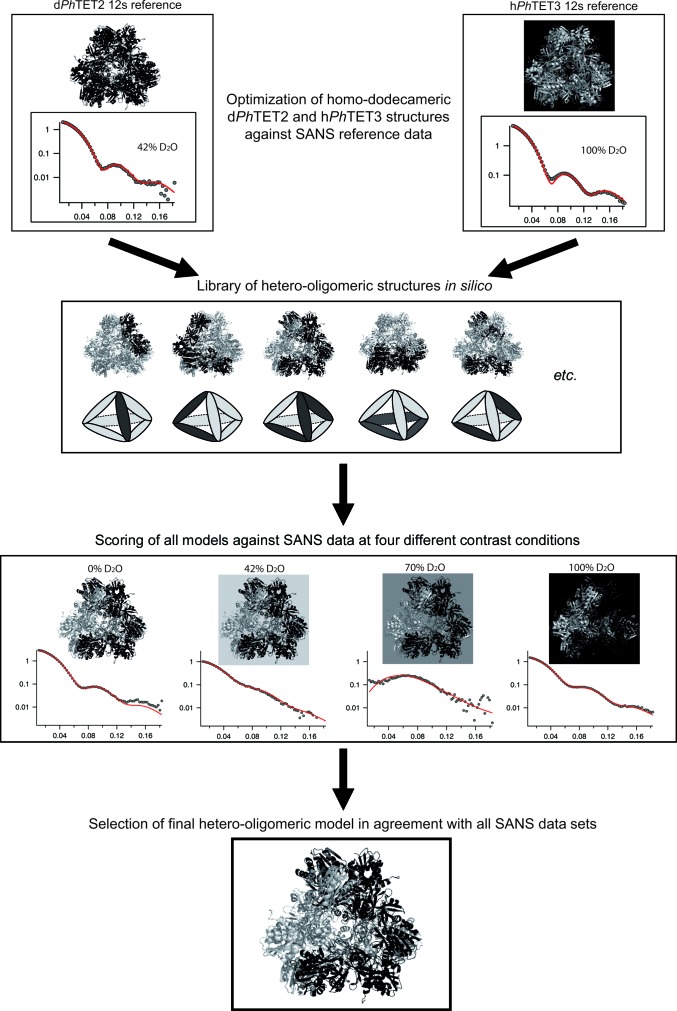
Workflow of the SANS approach adopted to determine the quaternary arrangement of deuterated *Ph*TET2 (black) and hydrogenated *Ph*TET3 (grey) building blocks within the heterododecameric particles in solution.

After the size-exclusion step of the purification, dodecameric d*Ph*TET2 and h*Ph*TET3 samples were dialyzed against a de-oligomerization buffer (50 m*M* CAPS, 20 m*M* NaCl, 20 m*M* EDTA pH 10). Aliquots of each sample were analyzed by size exclusion on a Superose 6 column (GE Healthcare) and on native polyacrylamide gels to control the oligomeric state of the samples after dialysis. According to the size-exclusion chromatogram (Supplementary Fig. S1^1^), both contained mostly TET dimers; the d*Ph*TET2 sample contained less than 4% monomers and the h*Ph*TET3 sample contained approximately 30% monomers. Therefore, less than 4% of the dimers present in solution can be d*Ph*TET2–h*Ph*TET3 heterodimers and such building blocks were therefore discarded in the modelling process, which was exclusively based on homodimeric building blocks (Figs. 1 ▶ and 2 ▶).

The de-oligomerized d*Ph*TET2 and h*Ph*TET3 samples were mixed together and dialyzed against re-oligomerization buffer (20 m*M* Tris–HCl, 150 m*M* NaCl, 2 m*M* CoCl_2_ pH 7.5). The re-oligomerized sample was analyzed on a Resource Q ion-exchange column (GE Healthcare) equilibrated in 150 m*M* NaCl, 20 m*M* Tris–HCl pH 7.5. After washing with 3 CV of the equilibration buffer, bound proteins were eluted at 4 ml min^−1^ with a 20 CV linear salt gradient (0.15–0.45 *M* NaCl in 20 m*M* Tris–HCl pH 7.5). Four peaks were obtained, two of which were predominant and contained a sufficient amount of protein for further characterization by SANS. According to their order of elution during the ion-exchange chromatography step, they were named peak 1 and peak 2. To increase the monodispersity of the samples, they were loaded onto a 1 ml Mono Q column (GE Healthcare) equilibrated in 150 m*M* NaCl, 20 m*M* Tris–HCl pH 7.5. After washing with 3 CV of the equilibration buffer, bound proteins were eluted at 1.5 ml min^−1^ with a 30 CV linear salt gradient (0.15–0.45 *M* NaCl in 20 m*M* Tris–HCl pH 7.5). They were then analyzed by size-exclusion chromatography (Superose 6) to control their final oligomerization state and both were exclusively do­decameric (Supplementary Fig. S10).

### 2.3. SANS sample preparation, experimental details and data reduction   

The samples corresponding to each peak from the final size-exclusion chromatography were split into two: one half was conserved in 100% H_2_O and the other half was equilibrated in 100% D_2_O. Buffer exchange (20 m*M* Tris–HCl, 150 m*M* NaCl pH 7.5 in 100% D_2_O or 100% H_2_O) was performed by dialysis overnight at 4°C. The D_2_O ratio of the different samples was then obtained by mixing the two solutions. 200 µl of protein solution was prepared at a concentration of 4.5 mg ml^−1^ at the following D_2_O ratios: 0, 42, 70 and 100% D_2_O. The D_2_O ratios were validated *via* their neutron transmission values. Apart from the 42% sample of peak 2, which was actually at 49% D_2_O, all samples were at the nominal ratio. Homododecameric h*Ph*TET2 (42% D_2_O), d*Ph*TET2 (42 and 100% D_2_O) and h*Ph*TET3 (100% D_2_O) reference samples were prepared following the same protocol.

Protein concentrations were measured using the Bio-Rad protein-assay reagent (Bio-Rad) with bovine serum albumin as a standard. Correction factors were applied to pure *Ph*TET2 and *Ph*TET3 samples. These factors were calculated after determining the protein concentration of pure *Ph*TET2 and *Ph*TET3 samples by quantitative amino-acid analysis as described in Durá *et al.* (2009 ▶). For the concentration of pure hetero-oligomeric samples, a weighted correction factor was calculated taking into account the correction factors obtained for *Ph*TET2 and *Ph*TET3 and the number of monomers of these proteins in the heteromeric complexes. Therefore, all of the values reported in this paper refer to real protein concentrations. We estimate the accuracy of the concentration measurements to be about 30%.

SANS experiments were performed on the large dynamic range small-angle diffractometer D22 at the Institut Laue–Langevin (ILL), Grenoble, France. The incident wavelength was λ = 6 Å (Δλ/λ = 10%) at a single detector/collimator configuration (4 m/4 m) with a centred beam. All samples and buffers were prepared the night before the experiment as described above. The final sample volumes were adjusted to 160 µl and placed in Hellma 1 mm QS quartz cells. Boron, empty cell and a pure H_2_O sample were measured as references for the subsequent data treatment. In a first experiment, we verified the match points of d*Ph*TET2 in 100% D_2_O and h*Ph*TET2 in 42% D_2_O (Supplementary Fig. S9). The temperature of the remaining samples, including buffers and references, was increased in steps of 20°C from 20 to 80°C. Typical exposure times were 20 min for the reference samples, mixtures and buffers at each temperature step. In addition, the empty cell, H_2_O and boron were measured for 10 min at each temperature. As a result, all samples were kept at each temperature for 4 h. Transmissions were measured for 3 min at the lowest temperature (20°C). The two-dimensional data sets of all samples and buffers were reduced to one-dimensional curves using the standard ILL software (Gosh *et al.*, 2006 ▶). Buffer intensities were subtracted from the respective sample intensities using *PRIMUS* from the *ATSAS* program suite (Konarev *et al.*, 2003 ▶).

### 2.4. SANS data analysis and modelling   

The model-free parameters *I*(0) (intensity scattered in the forward direction) and *R*_g_ (radius of gyration) were extracted from the SANS curves using the Guinier approximation (Guinier, 1939 ▶; Table 1 ▶). Pair-distance distribution functions *p*(*r*) were extracted using *GNOM* (Svergun, 1992 ▶) by imposing *p*(*r* = 0) = *p*(*r* = *D*_max_) = 0. Calibration of the molecular mass of the particles in solution was performed against water (Jacrot & Zaccai, 1981 ▶). The solvent-excluded volumes and scattering lengths of d*Ph*TET2 and h*Ph*TET3 monomers were calculated based on their amino-acid sequences (Jacrot, 1976 ▶), measured *I*(0), protein concentrations, transmissions and quartz-cell path lengths (Supplementary Table S1).

*Ab initio* shapes of the *Ph*TET2 and *Ph*TET3 moieties within the heterododecamers (Supplementary Fig. S7) were calculated with *MONSA* (Svergun, 1999 ▶) using two phases (d*Ph*TET2 and h*Ph*TET3) according to the following parameters: monomer volumes *V_Ph_*_TET2_ = *V_Ph_*_TET3_ = 50 000 Å^3^ (6s:6s stoichiometry, where ‘s’ designates a single subunit, *i.e.* a monomer), *V_Ph_*_TET2_ = 66 700 Å^3^, *V_Ph_*_TET3_ = 33 300 Å^3^ (8s:4s stoichiometry); the contrasts of d*Ph*TET2 were 5.75, 3.34 (2.91) and −0.02 × 10^10^ cm^−2^ in 0, 42 (49) and 100% D_2_O and the contrasts of h*Ph*TET3 were 2.30, −0.12 (−0.53) and −3.48 × 10^10^ cm^−2^ in 0, 42 (49) and 100% D_2_O. Connectivity of the two phases was not imposed.

For the pseudo-atomic models, *Ph*TET2 and *Ph*TET3 dodecameric models were generated from the crystal structures (PDB entries 1y0r and 2wzn, respectively; Borissenko & Groll, 2005 ▶; Durá *et al.*, 2009 ▶). Indeed, both crystal structures present a monomer within the asymmetric unit, so generating the dodecameric models relied on only the same symmetry operators. Both dodecameric models were adjusted to yield best fits (Fig. 1 ▶, top; Supplementary Fig. S12) against the respective SANS curves of the homododecameric reference samples d*Ph*TET2 12s (42% D_2_O) and h*Ph*TET3 12s (100% D_2_O) using *CRYSON* (Svergun *et al.*, 1995 ▶, 1998 ▶). In particular, the missing parts of the respective crystal structures were added manually with *Coot* (Emsley & Cowtan, 2004 ▶). In the case of the *Ph*TET2 model, addition of the missing part of the N-terminus (residues 1–5) as well as the internal loop (residues 120–132) led to a good fit of the d*Ph*TET2 12 s SANS reference curve in 42% D_2_O (Fig. 1 ▶, top; Supplementary Fig. S12). As a basic unit, dimeric building blocks were chosen (in agreement with previous SAXS data; Appolaire *et al.*, 2013 ▶) and generated with the symmetry operator prior to generating the whole dodecameric model. In the case of *Ph*TET3, addition of the N-terminal part (residues 1–7) as well as the internal loop (residues 128–136) led to a non­satisfactory fit of the h*Ph*TET3 12s SANS reference curve in 100% D_2_O. Consequently, the *Ph*TET3 dimeric building bock was generated using a manual rigid body to slightly change the relative position of one monomer with respect to the second monomer within the dimeric building block. In this way and by adding the N-terminal part, a dodecameric model was generated with a reasonably good fit to the SANS reference curve (Fig. 1 ▶, top; Supplementary Fig. S12). An overview comparing both of the dodecameric TET particles before and after the modifications as well as with each other is provided in Supplementary Fig. S11.

**Figure 2:**
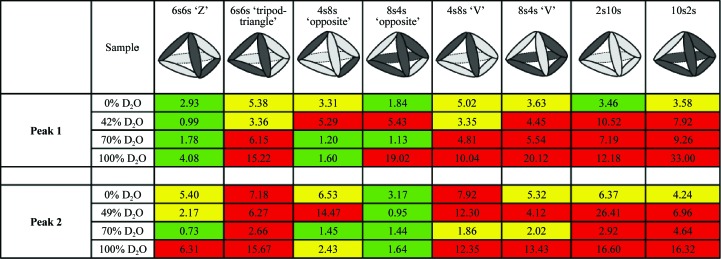
Overview of the fits of several homodimeric models against the four SANS contrast scattering curves. Deuterated *Ph*TET2 dimers are depicted by dark grey ellipsoids and hydrogenated *Ph*TET3 dimers are depicted as light grey ellipsoids. Green indicates excellent fits against SANS data at the respective H_2_O/D_2_O contrast, yellow moderate agreement and red strong disagreement. The numbers indicate the χ^2^ values obtained with *CRYSON*. Peak 1 can only be fitted by one model in a satisfactory way (d*Ph*TET2 6s:h*Ph*TET3 6s = ‘Z’), while peak 2 can only be fitted by a single, distinct model (d*Ph*TET2 8s:h*Ph*TET3 4s = ‘opposite’). All other dodecameric models based on homodimeric building blocks can readily be ruled out.

In order to facilitate the generation of heterododecameric hybrids, prior to dodecamer generation *Ph*TET2 and *Ph*TET3 dimeric models were superimposed in *Coot*. A library of heterododecameric hybrids was then created by editing the PDB files. The library exhaustively covered all dodecameric models based on homodimeric *Ph*TET2 and *Ph*TET3 building blocks (Fig. 2 ▶). All structures from the library were scored in a least χ^2^ fit against the SANS data in 0, 42 (49), 70 and 100% D_2_O for peak 1 and peak 2 using *CRYSON*. Fits were classified as excellent, moderate and bad for each model and contrast (Fig. 2 ▶) and were colour-coded green, yellow and red, respectively. A complete version of Fig. 2 ▶, including the individual fit curves, is provided as Supplementary Fig. S13. It illustrates that both χ^2^ values and visual criteria (good overall superposition of back-calculated and experimental curves) should be considered when discriminating between different models.

## 3. Results   

### 3.1. Purification of *Ph*TET2–*Ph*TET3 hetero-oligomers   

**Figure 3:**
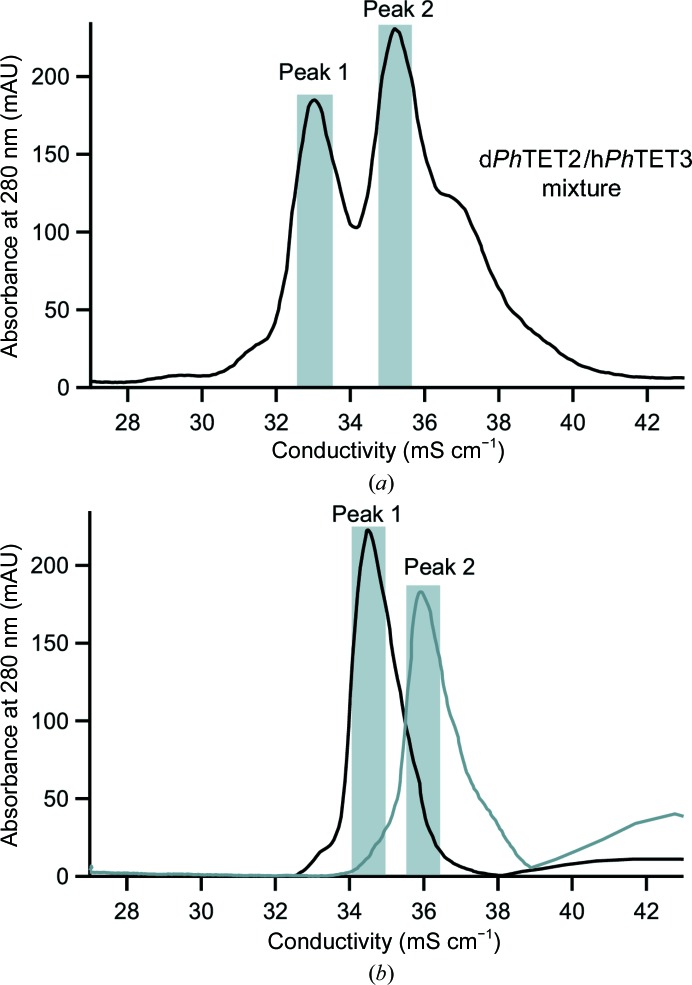
Purification of heterododecamers. The peaks are represented using the absorbance (*A*_280 nm_) as a function of the conductivity (mS cm^−1^). (*a*) Ion-exchange chromatography (Resource Q) of the total sample after the re-oligomerization process. The shaded areas correspond to the fractions grouped together for successive Mono Q purification. (*b*) Ion-exchange chromatography (Mono Q) of the two major peaks after the first Resource Q step. The shaded areas correspond to the fractions grouped together for size-exclusion chromatography on a Superose 6 column (Supplementary Fig. S10).

In the present study, we induced de-oligomerization of dodecameric deuterium-labelled *Ph*TET2 (‘d*Ph*TET2’) and dodecameric hydrogenated *Ph*TET3 (‘h*Ph*TET3’) into dimers. Subsequently, hetero-oligomers were generated by mixing the two dimer populations and inducing their re-oligomerization. Since *Ph*TET2 and *Ph*TET3 do not have the same external surface charges, their elution volumes are very different when analyzed appropriately by ion-exchange chromatography. Consequently, the different hetero-oligomeric assemblies can be separated by using an adequate ion-exchange chromatographic column run with a suitable salt gradient. After a first chromatographic step on a Resource Q column, two major (and several minor) peaks were observed (Fig. 3 ▶ *a*). In order to better separate the different hetero-oligomers, a second strong ion-exchange chromatography was performed on a Mono Q column (Fig. 3 ▶ *b*). (The fractions recovered from the Resource Q for the Mono Q are shaded in grey in Fig. 3 ▶ *a*.) The protein concentrations and monodispersity of the two major peaks (‘peak 1’ and ‘peak 2’) were high enough for subsequent SANS experiments.

### 3.2. SANS data reveal two extremely stable, heterododecameric d*Ph*TET2–h*Ph*TET3 architectures of different stoichiometry and quaternary structure   

**Figure 4:**
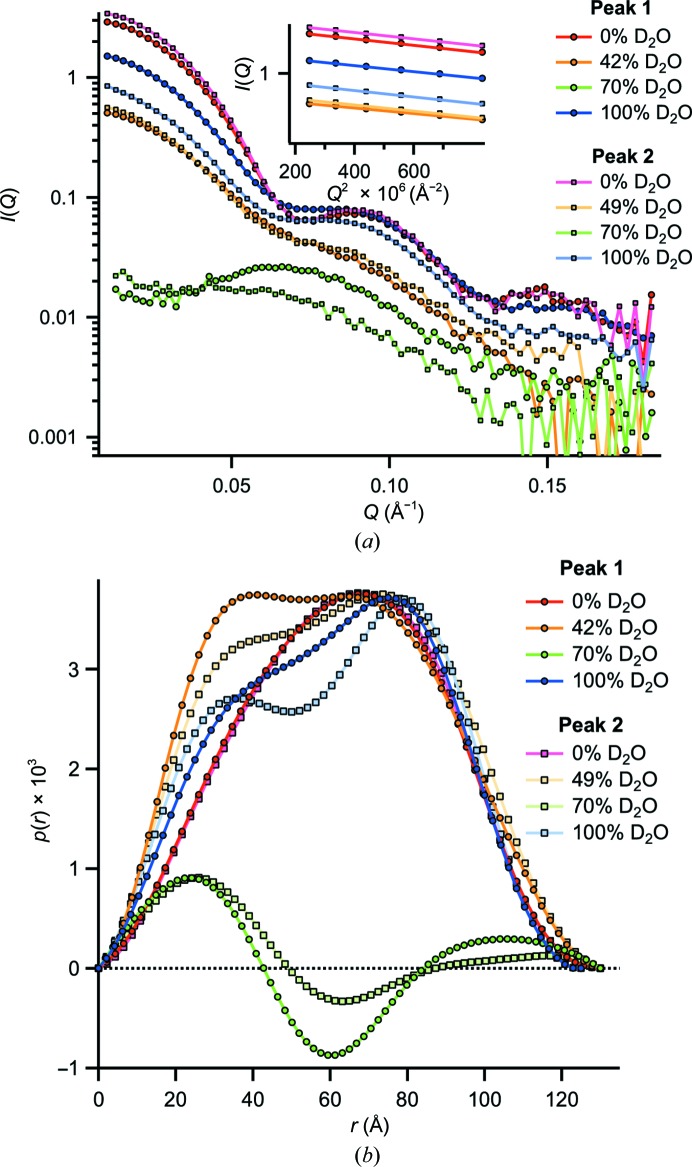
SANS curves and *p*(*r*) functions of peak 1 and peak 2. (*a*) SANS curves at four different contrast conditions at 20°C. All data sets are drawn without applying scaling. The total protein concentrations of all samples were identical (4.5 mg ml^−1^). Guinier fits to the 0, 42 (49) and 100% D_2_O data are shown as an inset (the 70% D_2_O data were not fitted using the Guinier approach owing to their elevated noise level). (*b*) Pair-distance distribution functions *p*(*r*) of the SANS data in arbitrary units generated with *GNOM* (Svergun, 1992 ▶). The two 0% D_2_O data sets are very similar (*cf.* Supplementary Fig. S6). The 0, 42, 49 and 100% D_2_O data are normalized to the second peak and the 70% D_2_O data to the first peak (using a different scaling factor for clarity).

The samples from both peaks yielded very specific and distinct SANS curves (Fig. 4 ▶ *a*) and model-free parameters *I*(0) and *R*_g_ (Table 1 ▶) at the four contrast conditions. At 0% D_2_O both resemble the homododecameric reference curves (Supplementary Fig. S6) most since both d*Ph*TET2 and h*Ph*TET3 moieties have positive contrast with respect to the solvent (Supplementary Fig. S4). The 70% D_2_O data have the lowest relative intensities since the hydrogenated and deuterated building blocks are of opposite contrast and result in a decrease in the overall scattered intensities. At 42 (49) and 100% D_2_O the scattered signals are almost exclusively owing to the deuterated and hydrogenated building blocks of the particles, respectively. Since small but significant variations of the curves at 0, 42 (49), 70 and 100% D_2_O are observed for both peaks, it can be concluded that the geometric arrangement of d*Ph*TET2 and h*Ph*TET3 building blocks are different within the particles constituting peak 1 and peak 2.

An intriguing feature is the evolution of the *I*(0) intensities as a function of contrast: while particle 2 (peak 2) scatters more strongly in the forward direction at 0, 42 and 70% D_2_O than particle 1 (peak 1), the situation is the inverse at 100% D_2_O. Since *I*(0), at a given total protein concentration, is proportional to the contrast integrated over the whole particle volume (Svergun & Koch, 2002 ▶), these findings indicate that particle 2 contains more deuterated building blocks than particle 1. Interestingly, these findings imply that at least two stoichiometrically different architectures are possible for heterododecameric TET particles *in vitro*. A calibration of the molecular masses against water (Jacrot & Zaccai, 1981 ▶) of the 0% D_2_O data yielded a d*Ph*TET2:h*Ph*TET3 stoichiometry of 6:6 for particle 1 and of 8:4 for particle 2 (Supplementary Table S1). Importantly, both hetero-oligomeric TET particles display extreme structural stability at high temperatures, with the SANS curves being virtually identical at 20 and 80°C (Supplementary Fig. S2). The same stability was found for mixtures of preformed homododecameric d*Ph*TET2 and h*Ph*TET2 particles for both the wild type and a pentamutant variant designed to weaken the oligomerization interface (Supplementary Fig. S3). Importantly, these data rule out a dynamic equilibrium between dodecameric assemblies and putative smaller oligomers in solution under our experimental conditions. The quality of the matching of hydrogenated and deuterated *Ph*TET2 was checked by measuring dodecameric h*Ph*TET2 and d*Ph*TET2 particles in 42 and 100% D_2_O, respectively. The results (Supplementary Fig. S9) indicated that the matching conditions were excellent, *i.e.* contributions from hydrogenated moieties can be neglected at 42% D_2_O and those of deuterated moieties at 100% D_2_O.

The pair-distance distribution functions *p*(*r*) extracted with *GNOM* (Svergun, 1992 ▶) from the SANS curves at the four contrast conditions revealed a wealth of real-space information on the relative positions of deuterated and hydrogenated building blocks within the two particles (Fig. 4 ▶ *b*). The back-fitted (regularized) scattering curves are shown in Supplementary Fig. S5 and show excellent agreement with the experimental data. An overview of the numerical values of *R*_g_ and *D*_max_ is provided in Table 1 ▶.

At 0% D_2_O both d*Ph*TET2 and h*Ph*TET3 building blocks have positive contrast (of different amplitude) with respect to the solvent (Supplementary Fig. S4). The *p*(*r*) functions from peak 1 and peak 2 are therefore almost identical, indicating that both particles possess a very similar overall shape (envelope) comparable to the d*Ph*TET2 and h*Ph*TET3 homododecameric references (Supplementary Fig. S6). The maxima of the leaning bell curves are shifted from the centre to larger distances and then drop rapidly to zero (a hallmark of hollow, globular particles; Koch *et al.*, 2003 ▶) and are in good agreement with the overall topology of dodecameric TET particles. The *D*_max_ of 130 Å is also in excellent agreement with the expected *Ph*TET dimensions from electron microscopy (Schoehn *et al.*, 2006 ▶).

At 42 (49)% D_2_O the *p*(*r*) functions are almost exclusively owing to deuterated moieties and therefore represent the internal arrangement of d*Ph*TET2 building blocks within the dodecameric edifices. In both cases a broadened central peak is observed. A tendency to split up into a bimodal pattern is observed for both particles: symmetric for particle 1 and asymmetric for particle 2. The observed bimodal *p*(*r*) patterns can be interpreted in terms of several compact, deuterated moieties positioned at a finite distance from each other, being either loosely connected or separated by small gaps. The 100% D_2_O data represent the complementary contrast condition, now with the hydrogenated building blocks (h*Ph*TET3) almost exclusively accounting for the *p*(*r*) patterns. Again, bimodal patterns are observed. Interestingly, the separation of both humps is more pronounced for particle 2, indicating that its respective h*Ph*TET3 dimers are, on average, more widely separated than those in particle 1.

At 70% D_2_O h*Ph*TET3 and d*Ph*TET2 building blocks have negative and positive contrast, respectively (Supplementary Fig. S4). Since *p*(*r*) functions contain products of pairs of volume elements weighted by their respective contrast, positive and negative regions can be observed in such cases (Glatter & Kratky, 1982 ▶): positive *p*(*r*) values occur if volume elements separated by the distance *r* are predominantly of identical contrast (positive–positive or negative–negative) and negative values are observed if volume elements of opposite contrast (positive–negative or negative–positive) dominate at this distance. The positive peak at short distances (*r* ≃ 20 Å) therefore belongs to volume elements within d*Ph*TET2 (or h*Ph*TET3) dimeric building blocks. At intermediate distances (*r* ≃ 60 Å) volume elements of opposite contrast (d*Ph*TET2–h*Ph*TET3 pairs) prevail, yielding a minimum, while at large distances (*r* ≃ 100–110 Å) pairs of volume elements with the same sign again dominate. Intriguingly, at 70% D_2_O the minimum at 60 Å is more pronounced for particle 1 than particle 2, indicating that in the former the d*Ph*TET2 and h*Ph*TET3 dimers are distributed more symmetrically (in number and geometry).

### 3.3. Pseudo-atomic models of *Ph*TET2–*Ph*TET3 complexes using SANS contrast-variation data   

**Figure 5:**
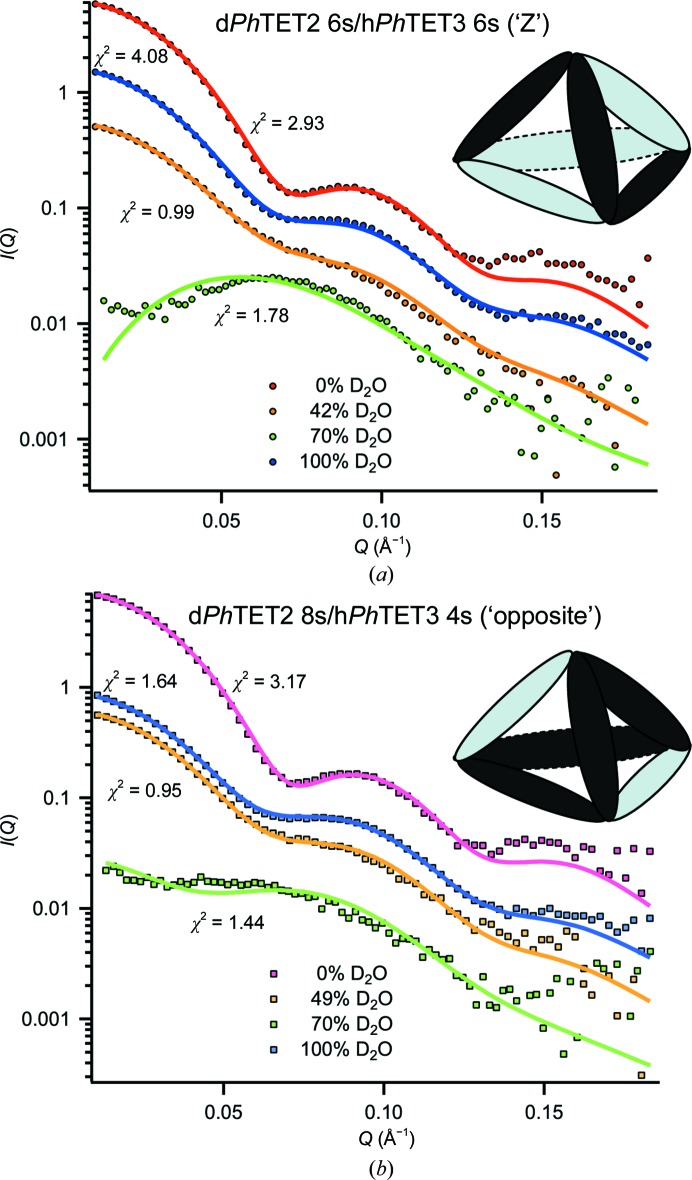
SANS data and best models for peak 1 and peak 2: the two architectures (‘Z’ and ‘opposite’) that are in best agreement with the SANS data of peak 1 (*a*) and peak 2 (*b*), along with their respective fits at four different contrasts. Elongated ellipsoids represent d*Ph*TET2 (dark grey) and h*Ph*TET3 (light grey) homodimers. Intriguingly, each tetrahedral apex brings together catalytic sites of different substrate specificity.

Fig. 1 ▶ illustrates the workflow of our approach to determine pseudo-atomic, quaternary structures of the heterododecameric *Ph*TET2–*Ph*TET3 particles. As a first step, homododecameric (‘12s’) reference structures of d*Ph*TET2 and h*Ph*TET3 were measured by SANS at 42 and 100% D_2_O, respectively. *Ph*TET2 and *Ph*TET3 dodecameric crystal structures (PDB entries 1y0r and 2wzn, respectively) were slightly modified to match these SANS reference data at ‘low resolution’ (Fig. 1 ▶, top) and were subsequently used to construct a library of heterododecameric models of variable stoichiometry and geometry (Figs. 1 ▶ and 2 ▶, Supplementary Fig. S13). All structures from the library were scored against the SANS data from peak 1 and peak 2 at 0, 42 (49), 70 and 100% D_2_O. A colour code (green, yellow and red) was used to design excellent, moderate and unacceptable fits. Only one architecture was able to satisfy all contrast conditions simultaneously for each peak and all others could readily be rejected (Fig. 2 ▶, Supplementary Fig. S13). Peak 1 was only fitted by a d*Ph*TET2 6s/h*Ph*TET3 6s architecture with the two hexamer (‘6s’) moieties arranged in an intertwined, double Z-shaped, clamp-like form (Fig. 5 ▶ *a*). Peak 2 was only fitted by a d*Ph*TET2 8s/h*Ph*TET3 4s model with the *Ph*TET3 moiety composed of two homodimers (‘4s’; four monomers) situated at opposite ridges of the dodecameric particle (Fig. 5 ▶ *b*).

In addition, and in complement, to the pseudo-atomic models, the multiphase *ab initio* program *MONSA* (Svergun, 1999 ▶) was used to calculate low-resolution envelopes of the *Ph*TET2 and *Ph*TET3 moieties within the heterododecameric particles of peak 1 and peak 2. A comparison of the low-resolution models with the pseudo-atomic models is shown in Supplementary Fig. S7. The *ab initio* shapes confirm, independently of the pseudo-atomic models, that *Ph*TET2 and *Ph*TET3 are organized as a symmetric architecture of two intertwined Z-shapes in particle 1 and that *Ph*TET2 is organized as two homodimers positioned on opposite ridges in particle 2.

### 3.4. The homododecameric *Ph*TET2 complex is extremely stable in solution and is not in dynamic equilibrium with free oligomeric forms of lower molecular mass   

As an additional stability control of the *Ph*TET quaternary structure and to test the presence of putative equilibria between preformed dodecameric particles with smaller oligomeric particles in solution, we measured mixtures of pre-formed homododecameric deuterated and hydrogenated *Ph*TET2 proteins (d*Ph*TET2 ‘12s’ and h*Ph*TET2 ‘12s’) by monitoring their SANS curves as a function of temperature and exposure time. The experiments were performed on both the wild-type protein and a pentamutant (R217S, R220S, F224S, H248S and I292A) with the mutations situated at the interface between the dimers and designed to slow the oligomerization process (Appolaire *et al.*, 2013 ▶). For the wild-type and the mutated *Ph*TET2, the following samples were prepared: h*Ph*TET2 (42% D_2_O), d*Ph*TET2 (42% D_2_O), h*Ph*TET2 (100% D_2_O) and d*Ph*TET2 (100% D_2_O). Different mixtures of the above samples were prepared by mixing 1:1 volume fractions of these solutions.

The SANS curves of isolated h*Ph*TET2 (100% D_2_O) and d*Ph*TET2 (42% D_2_O) reference particles (Supplementary Fig. S3) revealed the typical pattern of the dodecameric edifice as observed in previous SANS experiments (Durá *et al.*, 2009 ▶). Supplementary Fig. S3(*a*) shows a superposition of the scattering curves of wild-type h*Ph*TET2 in 100% D_2_O at *t* = 0 (20°C) and of a 1:1 mixture of d*Ph*TET2 and h*Ph*TET2 in 100% D_2_O after 4 h at 80°C, both normalized to h*Ph*TET2 concentration. Supplementary Fig. S3(*b*) shows the respective data sets of a 1:1 d*Ph*TET2/h*Ph*TET2 mixture at 42% D_2_O. Interestingly, the scattering patterns of both mixtures after 4 h at 80°C are identical to those of their respective dodecameric *Ph*TET2 references at 20°C regarding radii of gyration, position of the side maxima and minima and *I*(0) intensities. These very clear results indicate that no exchange of lower oligomeric building blocks between the hydrogenated and deuterated dodecamers had occurred in solution on the temperature and time scales of our SANS experiments. These experiments confirm the great stability of both the wild-type and pentamutant dodecameric particles once they are formed in solution.

## 4. Discussion   

Large and symmetric complexes of multiple copies of a single or a few similar building blocks play essential roles in the life cycle of biological cells (Matthews & Sunde, 2012 ▶; Griffin & Gerrard, 2012 ▶; Sauer & Baker, 2011 ▶; Snider & Houry, 2008 ▶; Marianayagam *et al.*, 2004 ▶). If crystal structures are not available, they represent a formidable challenge for many structural biology techniques: for NMR owing to their size and for SAXS or EM because it is very difficult to position individual subunits within the assembled complexes. The heterododecameric *Ph*TET2–*Ph*TET3 com­plexes studied here are highly symmetric and we applied a powerful combination of SANS, deuterium labelling and contrast variation to obtain unique insights into the oligomeric organization of their quaternary architectures. In contrast to EM or SAXS, the neutron scattering lengths for hydrogen and deuterium differ significantly and are of opposite sign (Jacrot, 1976 ▶). SANS can therefore focus specifically on D- or H-labelled partners within a reconstituted complex. Using this approach, we were able to eliminate a multitude of potential stoichiometries and geometric arrangements very efficiently and to clearly identify a single, specific architecture of the *Ph*TET2 and *Ph*TET3 moieties within the final heterododecameric complexes from peak 1 and peak 2 (Fig. 2 ▶, Supplementary Fig. S13).

**Figure 6:**
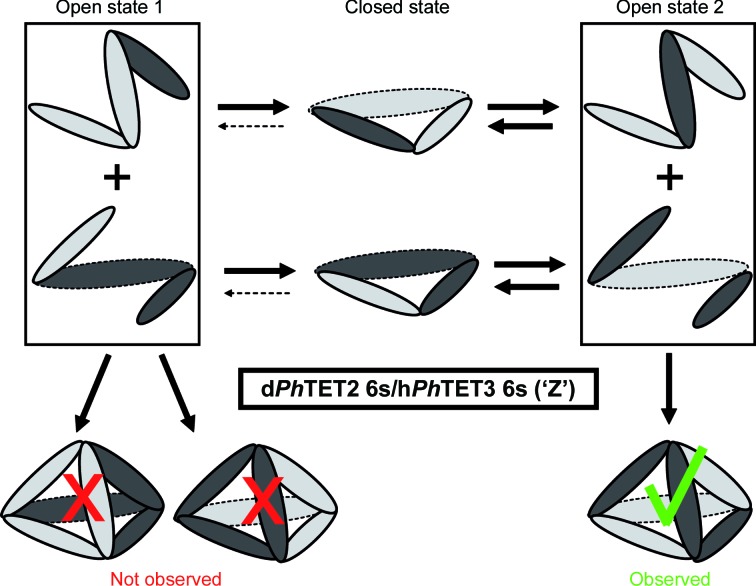
Assembly pathway for the ‘Z’ d*Ph*TET2 6s:h*Ph*TET3 6s heterododecamer (peak 1): the observation of a specific internal hexamer–hexamer topology by SANS, together with the exclusion of alternative topologies, points to a specific assembly pathway involving intertwined heterohexamers. Thick continuous and thin broken arrows represent strong and weak pathways for the dynamic equilibrium between respective states. An alternative assembly mode is presented in Supplementary Fig. S8.

Previously, a combination of site-directed mutagenesis, SAXS, AUC and EM allowed us to propose an assembly mechanism in which three dimers associate into a hexameric precursor prior to the formation of TET dodecamers (Appolaire *et al.*, 2013 ▶). This model accounts well for the very limited number of hetero-oligomeric TET complexes that we have identified in the present work by ion-exchange chromatography after metal-induced re-oligomerization of a mixture of *Ph*TET2 and *Ph*TET3 dimers (Fig. 3 ▶). This strategy was possible because the *Ph*TET2 and *Ph*TET3 partners behave differently on strong ion-exchange chromatography, allowing the separation of different hetero-oligomeric complexes as a function of their *Ph*TET2:*Ph*TET3 ratio. The SANS study described here provides direct insight into this assembly model. Indeed, the topology of the *Ph*TET2 and *Ph*TET3 moieties within the two main hetero-oligomeric species observed are in excellent agreement with an assembly process that involves a hexameric intermediate (Fig. 6 ▶, Supplementary Fig. S8), as suggested by a previous study combining SAXS, EM and AUC (Appolaire *et al.*, 2013 ▶).

Each of the TET apices defines a catalytic subcompartment in which three active sites are located on the same plane. Moreover, a nonprocessive mode of action has been reported for the three TET proteins from *P. horikoshii* (Durá *et al.*, 2005 ▶, 2009 ▶; Schoehn *et al.*, 2006 ▶). Therefore, after the cleavage of the N-terminal amino acid the polypeptide substrate is released from the active site and is free to interact with one of the two other catalytic pockets. In the present study we showed that, unexpectedly, TET dodecameric quaternary structures follow a highly organized pathway when self-assembling *in vitro*. This results in a limited number of architectures with a single type of apex composition, systematically combining *Ph*TET2 and *Ph*TET3 active sites with different and complementary substrate specificities (Fig. 6 ▶), suggesting that the highly controlled oligomerization process of the *Ph*TET2–*Ph*TET3 complex is to optimize its peptide-degradation efficiency.

Finally, our work shows that SANS experiments allow the respective positions of the different subunits to be very efficiently specified in the case of a large symmetrical pseudo-homomeric complex, information that is very difficult, if not impossible, to obtain using most biophysical techniques. In conclusion, such a SANS approach could be very efficient to better understand the assembly pathway of other large, symmetric, pseudo-homomeric complexes such as the proteasome (Sahara *et al.*, 2014 ▶), chaperonins (Kim *et al.*, 2013 ▶) or AAA-ATPases (Bar-Nun & Glickman, 2012 ▶). Our structural insights raise intriguing questions on the underlying regulatory mechanisms controlling the assembly process of such biomacromolecular complexes, a field of research that has remained poorly explored to date but might offer important perspectives for biomedical research and drug development in the future.

## Supplementary Material

Supplementary Figs. S1-S12 and Supplementary Table S1.. DOI: 10.1107/S1399004714018446/wa5075sup1.pdf

High resolution Supplementary Fig. S13.. DOI: 10.1107/S1399004714018446/wa5075sup2.png

## Figures and Tables

**Table 1:** Model-free SANS parameters of the heterododecameric and homododecameric complexes at 20C The forward scattered intensities *I*(0) and radii of gyration *R*_g_ extracted from the Guinier ranges (*Q*_min_ *R*_g_ *Q*_max_ *R*_g_; fits are shown as an inset in Fig. 4 ▶ *a*) are shown in comparison to the values extracted by indirect Fourier transform using *GNOM* (Svergun, 1992 ▶). The values extracted from both approaches are in very good agreement. The Guinier parameters were not determined for the 70% D_2_O data sets owing to high noise levels. The two *GNOM* *R*_g_ values represent the direct/indirect-space values, respectively.

Sample	*I*(0) (Guinier)	*R*_g_ (Guinier) ()	*Q*_min_ *R*_g_ *Q*_max_ *R*_g_	*R*_g_ (*GNOM*) ()	*D*_max_ (*GNOM*) ()
Peak 1 (0% D_2_O)	3.28 0.01	48.6 0.2	0.771.40	47.8/48.0	130 5
Peak 1 (42% D_2_O)	0.56 0.01	45.4 0.7	0.721.31	45.8/45.8	130 5
Peak 1 (70% D_2_O)	N.D.	N.D.	N.D.	34.4/38.1	130 5
Peak 1 (100% D_2_O)	1.66 0.01	47.6 0.2	0.751.37	47.0/47.2	125 5
Peak 2 (0% D_2_O)	3.84 0.02	48.6 0.2	0.771.40	48.1/48.3	130 5
Peak 2 (49% D_2_O)	0.62 0.01	47.7 0.6	0.751.38	47.5/47.6	130 5
Peak 2 (70% D_2_O)	N.D.	N.D.	N.D.	24.3/25.6	130 5
Peak 2 (100% D_2_O)	0.90 0.01	48.6 0.3	0.651.40	47.3/47.5	125 5
d*Ph*TET2 12s (42% D_2_O)	2.32 0.01	49.6 0.3	0.781.43	48.4/48.6	130 5
h*Ph*TET3 12s (100% D_2_O)	5.28 0.02	50.9 0.2	0.681.33	49.1/49.3	135 5

## Footnotes

1 Supporting information has been deposited in the IUCr electronic archive (Reference: WA5075).
