# Supplementary material for: Small-angle neutron scattering reveals the assembly mode and oligomeric architecture of TET, a large, dodecameric aminopeptidase
Source: Acta Crystallogr D Biol Crystallogr. 2014 Oct 23;70(Pt 11):2983–93. doi: 10.1107/S1399004714018446 (PMC4220976; doi:10.1107/S1399004714018446)
Supplement: Supplementary file 1 [file d-70-02983-sup1.pdf]

Supplementary information

Small angle neutron scattering reveals the assembling mode and oligomeric organization of TET, a large dodecameric aminopeptidase

Alexandre Appolaire, Eric Girard, Matteo Colombo, M. Asunción Durá, Martine Moulin, Michael Härtlein, Bruno Franzetti and Frank Gabel

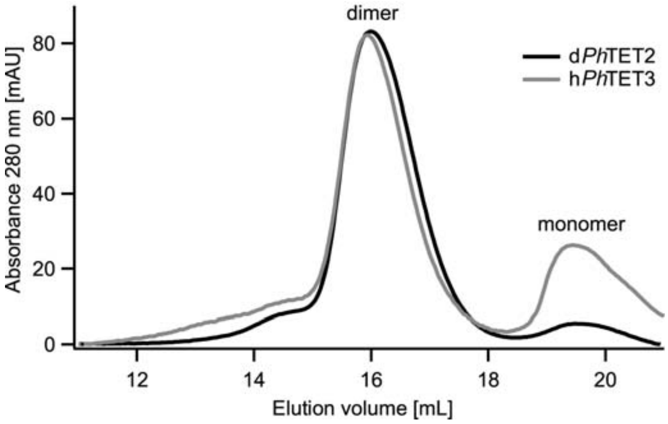

Fig. S1: **Elution profile on a size exclusion column (Superose 6) of the d*Ph*TET2 and h*Ph*TET3 samples immediately after the de-oligomerization step.** h*Ph*TET3 contains approximately 30% of monomers, while d*Ph*TET2 yielded less than 4% of monomers, limiting the overall probability to find hetero-dimers to a maximum of 4%.

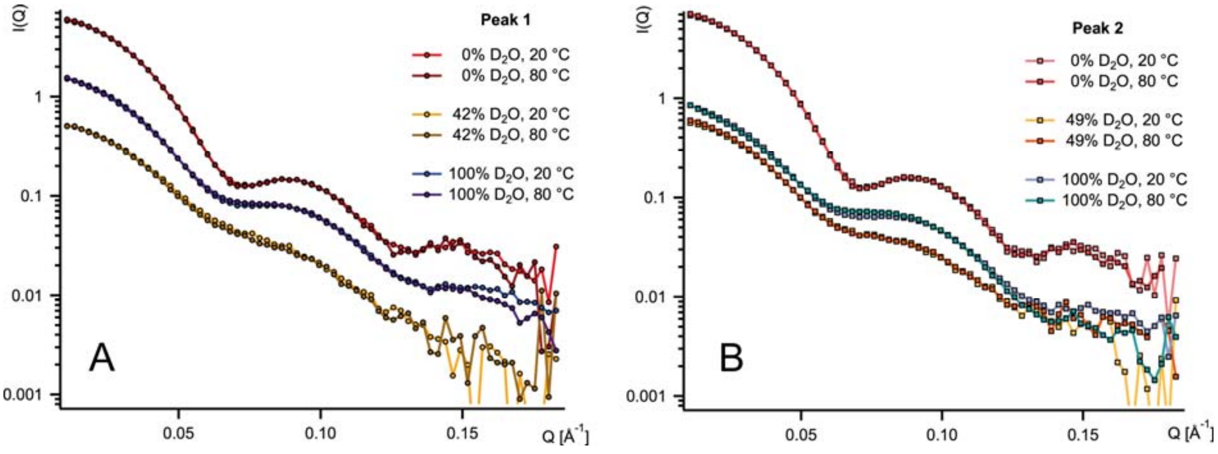

Fig. S2: Temperature dependence of the hetero-dodecamer SANS curves. Experimental SANS data of Peak 1 (A) and Peak 2 (B) at three contrast conditions at 20°C and after four hours at 80°C. No major changes in shape or intensity were observed between the respective datasets, indicating that both the oligomeric state and the shape of the two hetero-dodecameric complexes are stable over several hours at 80°C. A slight flattening of the first minimum is observed at 80°C, possibly due to an increased conformational flexibility of loops, C- and N-terminal parts at higher temperatures. 70% D_2O datasets were not recorded at 80 °C.

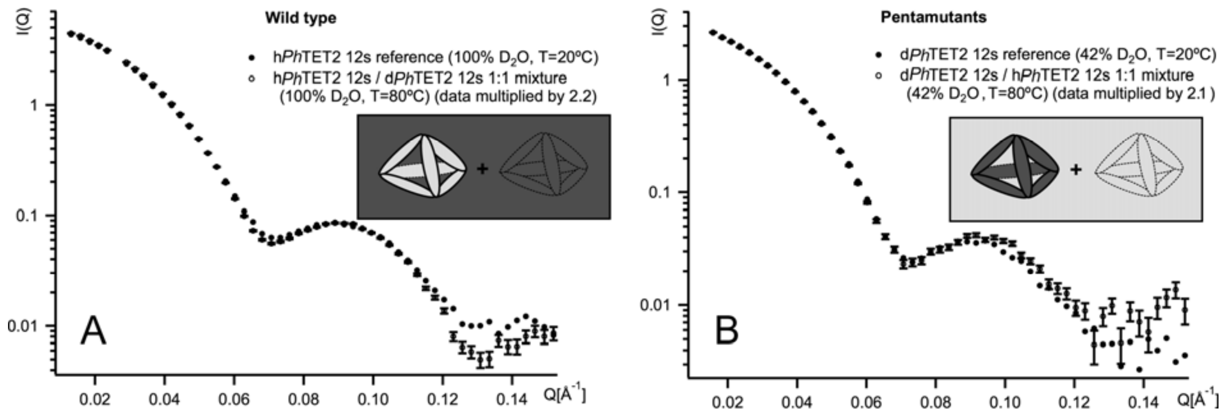

Fig S3. Stability of h*Ph*TET2 and d*Ph*TET2 homo-dodecamers (“12s”). A) Superposition of SANS curves from dodecameric wild type h*Ph*TET2 alone in solution at 20°C (reference) and a 1:1 mixture of h*Ph*TET2 and d*Ph*TET2 at 80°C, both in 100% D_2O. The signal of the mixture was scaled by a factor of 2.2 which corresponds well to the factor expected from the dilution. Pentamutant h*Ph*TET2 samples displayed an equivalent behavior. B) Superposition of SANS curves from pentamutant dodecameric d*Ph*TET2 alone in solution at 20°C (reference) and a 1:1 mixture of h*Ph*TET2 and d*Ph*TET2 pentamutants at 80°C, both in 42% D_2O. The signal of the mixture was scaled by a factor of 2.1 which agrees well with the factor expected from the dilution. Wild-type d*Ph*TET2 samples displayed an equivalent behavior.

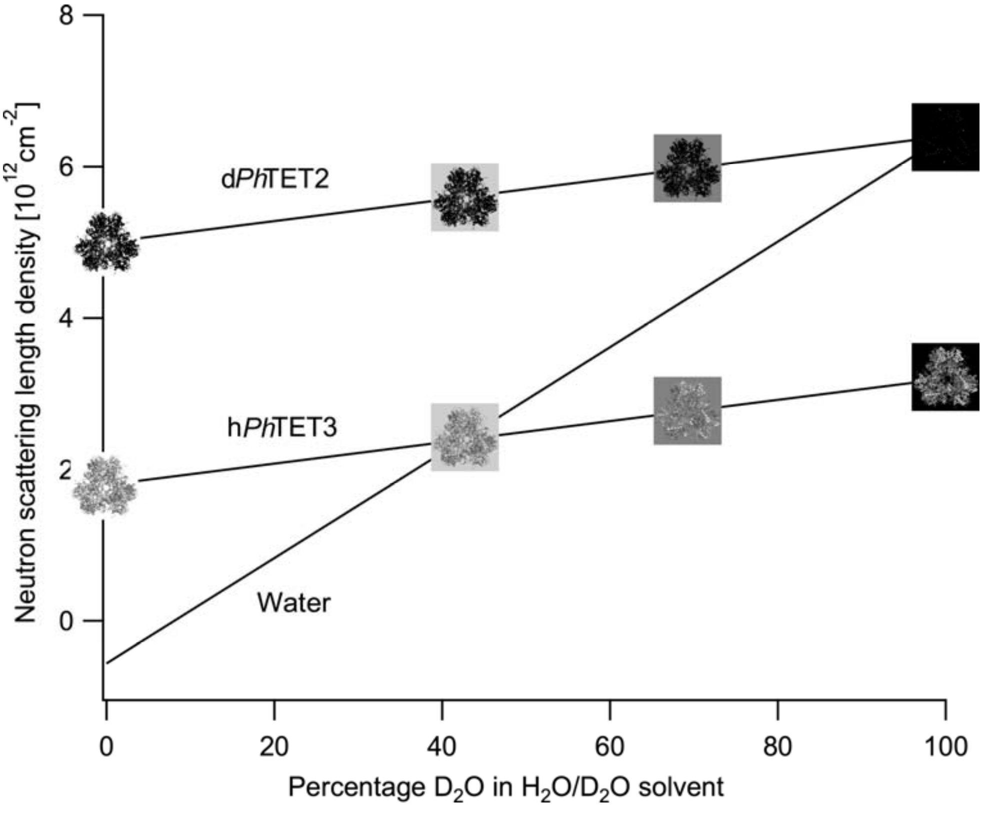

Fig. S4: Representation of the contrast conditions for d*Ph*TET2 and h*Ph*TET3. Neutron scattering length densities of solvent, d*Ph*TET2 and h*Ph*TET3. d*Ph*TET2 (black) and h*Ph*TET3 (gray) are depicted in front of a white, gray, dark gray or black background representing 0, 42, 70 and 100% D_2O in the solvent, respectively (This color code is applied throughout the present work). *Ph*TET particles that have positive contrast are above the water solvent line, those with negative contrast are below.

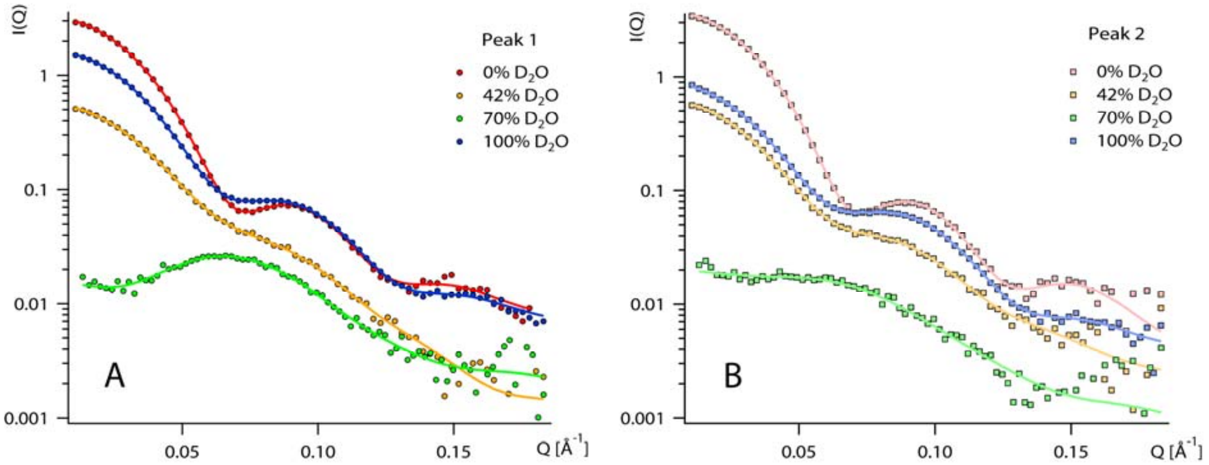

Fig. S5: GNOM fits of the SANS data at 20°C. Experimental SANS data of Peak 1 (A) and Peak 2 (B) at the four contrast conditions (filled symbols). The continuous lines of identical colors show the respective regularized curves calculated by the program GNOM. These regularizations were used to extract the *p*(*r*) functions shown in Fig. 2. In all cases (apart from the 70% D_2O datasets) the fits were judged as “excellent” by GNOM (“reasonable” for the 70% D_2O data).

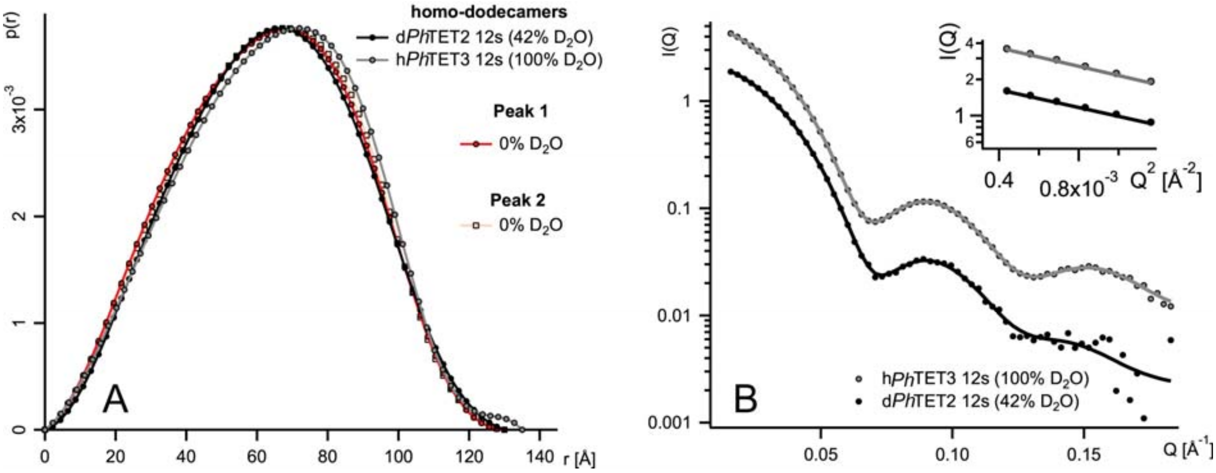

Fig. S6: *p*(*r*) functions of hetero- and homo-oligomers. A) Comparison of pair-distance distribution functions of the homo-dodecameric particles with respect to the hetero-dodecamers. B) GNOM back-calculated scattering curves against the respective experimental SANS curves for the homo-dodecameric particles. The respective Guinier fits are shown as an inset. The agreement is excellent. All datasets shown were recorded at 20°C.

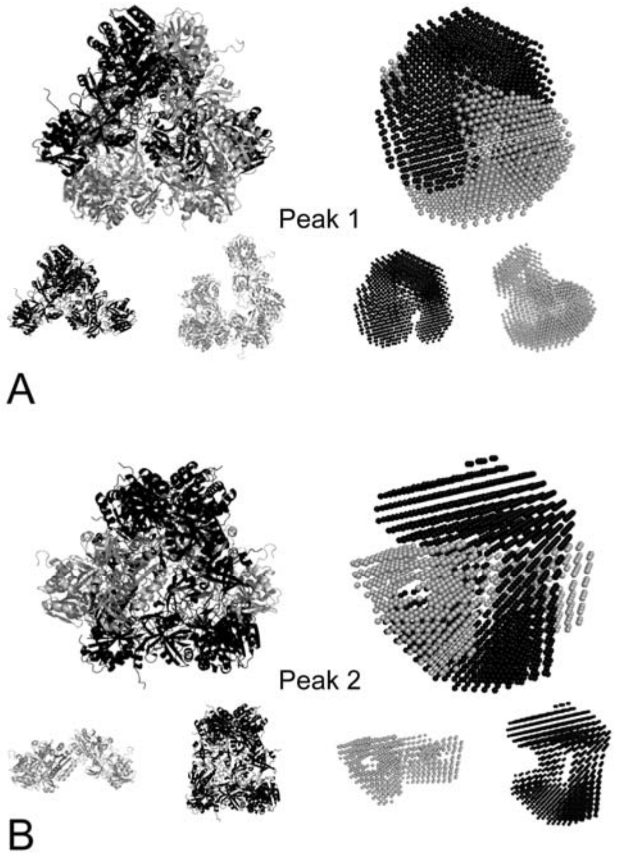

Fig. S7: A) Comparison of the final quasi-atomic model of Peak 1 and its low-resolution reconstitution from MONSA. The individual d*Ph*TET2 (black) and h*Ph*TET3 (gray) moieties are shown as an inset at the bottom. **B) Comparison of the final quasi-atomic model of Peak 2 and its low-resolution reconstitution from MONSA.** The individual d*Ph*TET2 (black) and h*Ph*TET3 (gray) moieties are shown as an inset at the bottom.

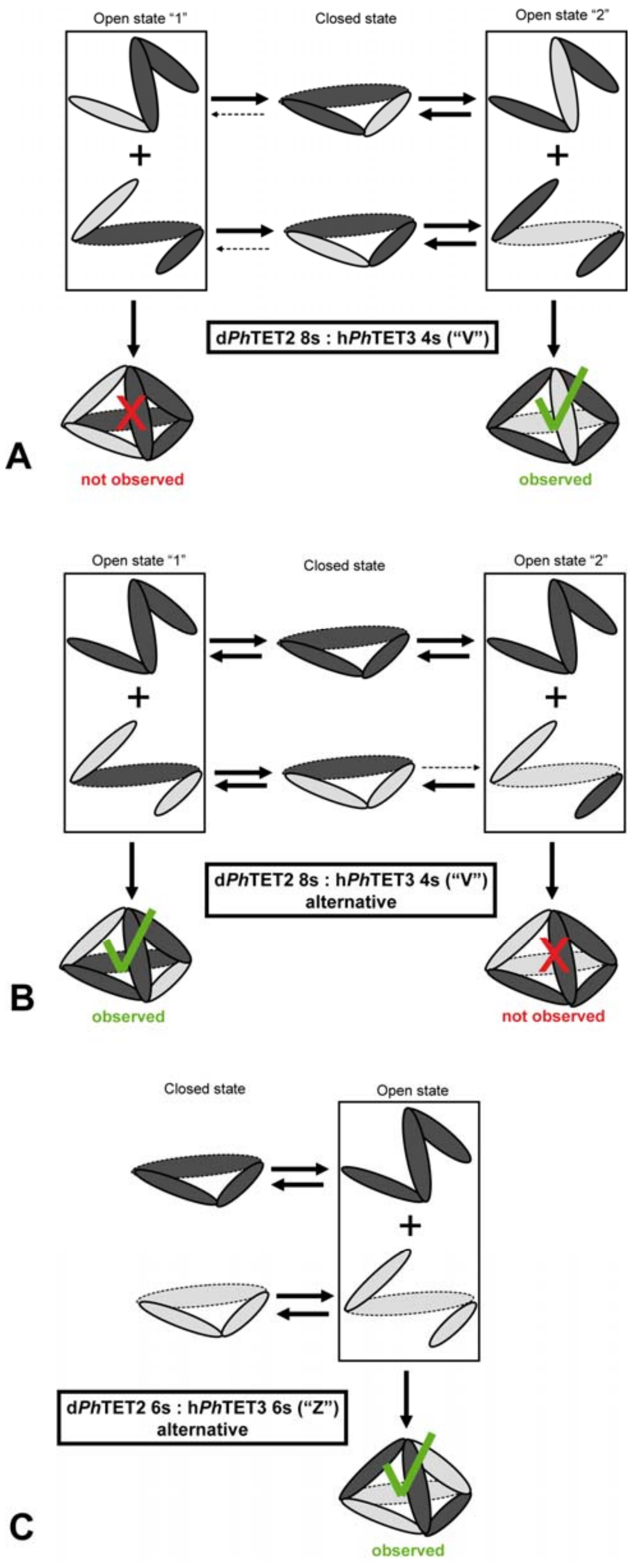

Fig. S8: Assembling pathways for the "opposite" 8s4s (Peak 2) and the "Z" 6s6s (Peak 1) hetero-dodecamers: A) and B) all possible assembling modes for a hetero-complex consisting of four d*Ph*TET2 and two h*Ph*TET3 dimers based on intermediate hexamers. The observed forms (two opposite h*Ph*TET3 dimers) display mixed *Ph*TET2:*Ph*TET3 apices and therefore heterogeneous catalytic chambers at each apex. C) An alternative pathway for the "Z" 6s6s particle from Fig. 5 which yields the same final architecture. In all cases, bold, continuous and broken fine arrows represent strong and weak pathways for the dynamic equilibrium between respective states.

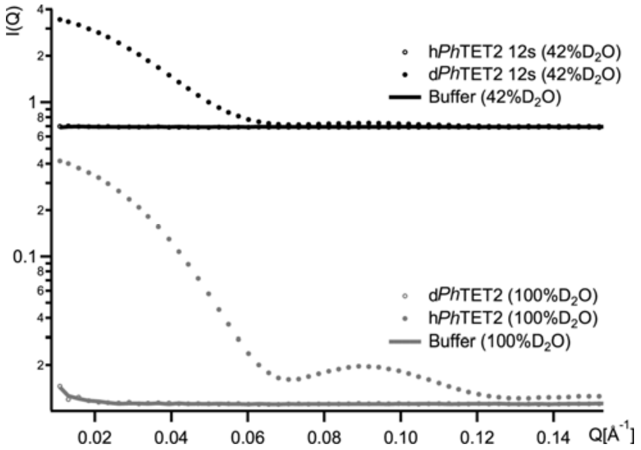

Fig. S9: Experimental validation of SANS contrast matching conditions at 20°C. SANS curves of mono-dodecameric h*Ph*TET2 and d*Ph*TET2 (wild-type) in 42 and 100% D_2O (prior to buffer subtraction) and the respective buffers. The upper part of the figure illustrates clearly that the hydrogenated partner (h*Ph*TET2) is perfectly matched at 42% D_2O and that the signal at this contrast point is dominated by the deuterated partner (d*Ph*TET2). The lower part demonstrates that the inverse situation is true at 100% D_2O (the latter data have been shifted for clarity).

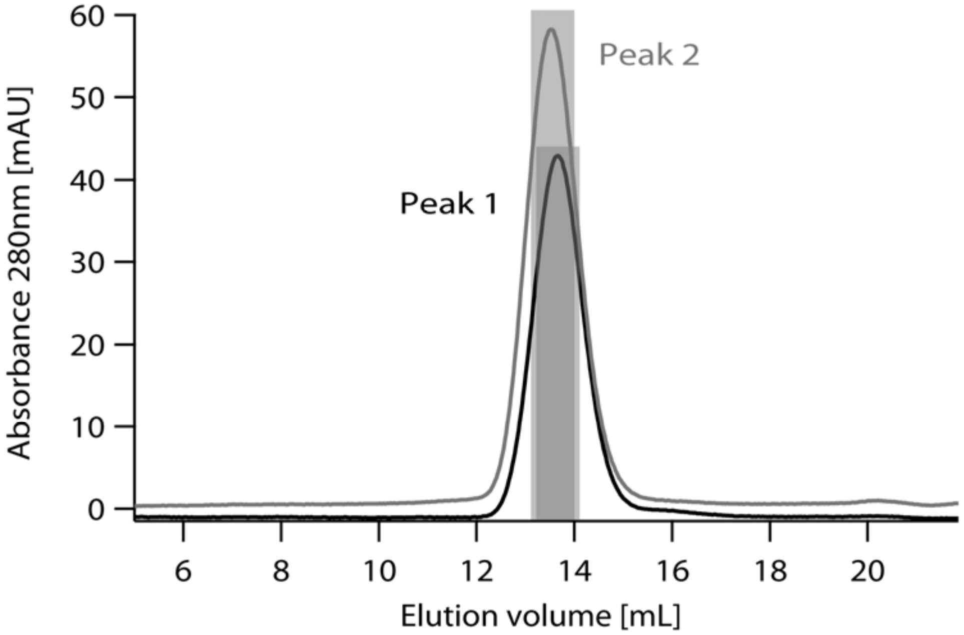

Fig. S10: **Elution profile on a size-exclusion column (Superose 6) of the Mono Q purified peaks 1 and 2.** The fractions shaded in gray were grouped together to constitute the samples measured by SANS.

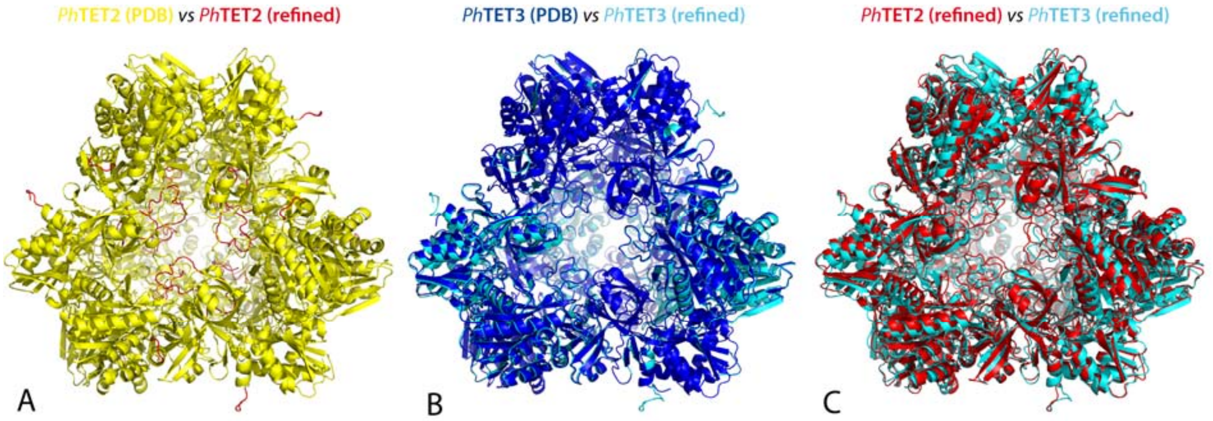

Fig. S11: **Comparison of *Ph*TET2 and *Ph*TET3 homo-dodecameric reference particles for SANS.** A: Original *Ph*TET2 crystal structure (PDB entry 2Y0R) (yellow). The N-terminus (residues 1-5) and the inner loop (residues 120-132) that have been added to optimize the fit against the SANS reference data (d*Ph*TET2 12s in 42% D_2O) are shown in red. B: Original *Ph*TET3 crystal structure (PDB entry 2WZN) (blue). The model refined by rigid-body motions to optimize the fit against the SANS reference data (h*Ph*TET3 12s in 100% D_2O) is shown in cyan. C: Superposition of the refined *Ph*TET2 (red) and *Ph*TET3 (cyan) models.

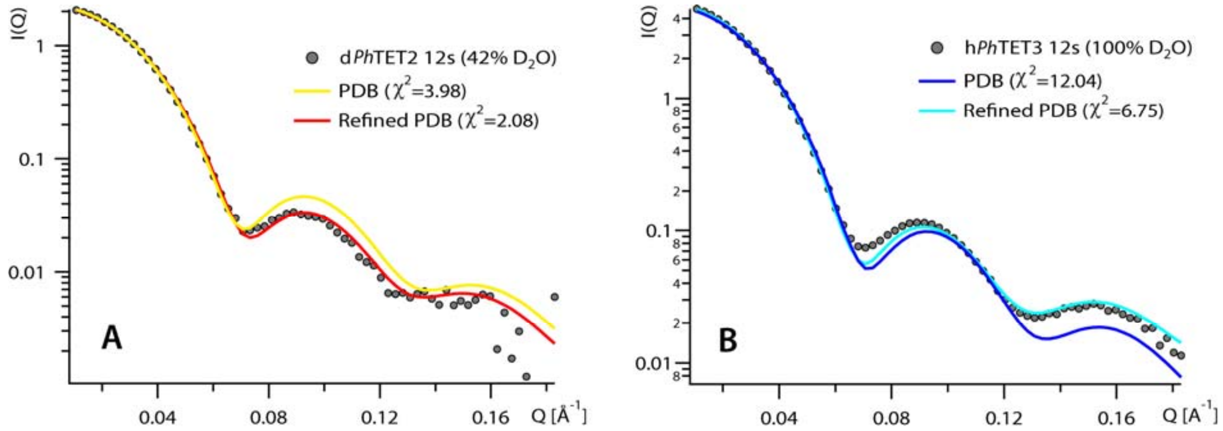

Fig. S12: **Comparison of fits of *Ph*TET2 and *Ph*TET3 homo-dodecameric reference particles against SANS data (same colour code as in Fig. S11).** A: Original *Ph*TET2 crystal structure (PDB entry 2Y0R) (yellow) and refined model (red) against the SANS reference data (d*Ph*TET2 12s in 42% D_2O). B: Original *Ph*TET3 crystal structure (PDB entry 2WZN) (blue) and the refined model (cyan) against the SANS reference data (h*Ph*TET3 12s in 100% D_2O).

	0% D_2O	42% D_2O	49% D_2O	100% D_2O
h*Ph*TET3 12s	1.06	0.0023	0.066	**4.19**
h*Ph*TET3 10s / d*Ph*TET2 2s	1.66	0.06	0.0016	2.90
h*Ph*TET3 8s / d*Ph*TET2 4s	2.39	0.30	0.11	1.84
h*Ph*TET3 6s / d*Ph*TET2 6s	**3.26**	**0.71**	0.40	**1.02**
h*Ph*TET3 4s / d*Ph*TET2 8s	**4.26**	1.30	**0.86**	**0.47**
h*Ph*TET3 2s / d*Ph*TET2 10s	5.39	2.07	1.50	0.10
d*Ph*TET2 12s	6.65	**3.01**	2.31	0.001
**"Peak1" (experimental)**	**3.28**	**0.56**	**-**	**1.66**
**"Peak2" (experimental)**	**3.84**	**-**	**0.62**	**0.90**
**h*Ph*TET3 12s (experimental)**	-	-	-	**5.28**
**d*Ph*TET2 12s (experimental)**	-	**2.32**	-	-

Table S1: Comparison of measured with calculated *I*(0) intensities. The measured *I*(0) intensites are the same as the ones in Table 1 of the main document. The theoretical ones (first seven data lines) were calculated according to (Jacrot & Zaccai 1981), using the following equation:

$$I(0) = f\,\frac{4\pi T_s}{1-T} I_{inc}(0) \frac{C}{M_r} N_A t 10^{-3} \left(\sum_i b_i - \rho_s V \right)^2 \qquad \text{(Suppl. Eq. 1)}$$

f=0.8 is correction factor taking the anisotropic scattering of the incoherent background into account, T_s and T are the sample and H_2O transmissions, respectively, $I_{inc}(0)$ is the incoherent water background at zero angle, C=4.5 mg/ml are the sample concentrations, N_A is Avogardo's constant, t=0.1cm is the sample cell pathlength. $\sum_i b_i$ and V are summed protein scattering lengths and solvent-excluded volumes, calculated from their amino acid sequences (Jacrot 1976) and ρ_s is the solvent scattering length density at the respective contrast conditions. $\sum_i b_i$ were calculated assuming that all exchangeable hydrogens exchange. The precision of the theoretical intensities is limited by the precision of the sample concentrations (determined by Bradford in our case) but depends also strongly on solvent-excluded volumes and the fraction of exchangeable hydrogens that is assumed. We estimate the overall precision with ± 30%. As can be seen from the two reference data sets (h*Ph*TET3 12s and d*Ph*TET2 12s), the discrapancy between experimental and theoretical values can be both positive or negative.
