# Supplementary figures and images for: Small-angle neutron scattering reveals the assembly mode and oligomeric architecture of TET, a large, dodecameric aminopeptidase
Source: Acta Crystallogr D Biol Crystallogr. 2014 Oct 23;70(Pt 11):2983–93. doi: 10.1107/S1399004714018446 (PMC4220976; doi:10.1107/S1399004714018446)

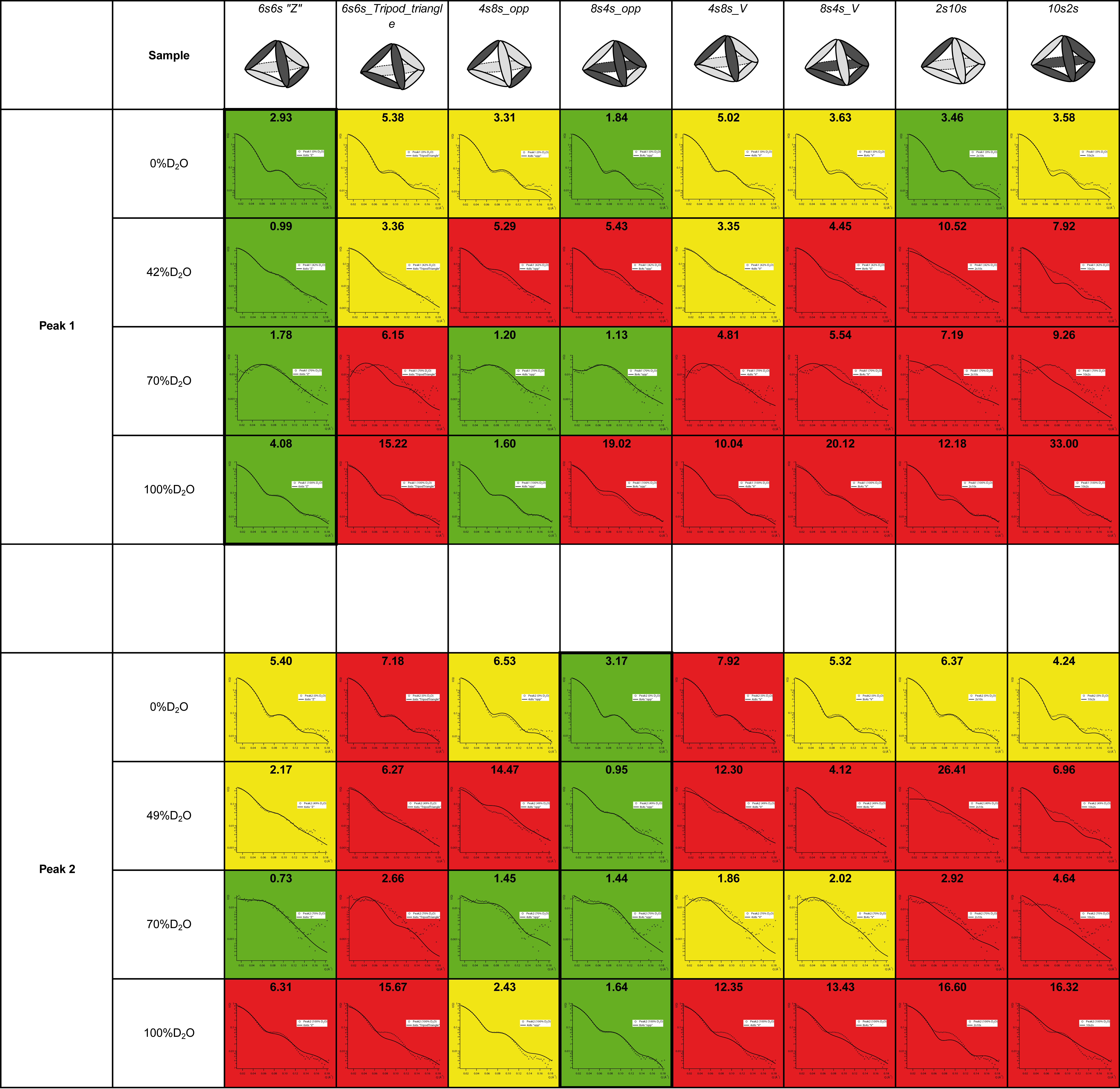

Supplement: Supplementary file 2 [file d-70-02983-sup2.png]
